# A Bayesian probabilistic analysis of the use of English modal verbs in L2 writing: Focusing on L1 influence and topic effects

**DOI:** 10.1016/j.heliyon.2024.e28701

**Published:** 2024-03-27

**Authors:** Fatih Ünal Bozdağ, Gareth Morris, Junhua Mo

**Affiliations:** a Faculty of Humanities and Social Sciences, Osmaniye Korkut Ata University, Fakiusagi Mah, 80000, Merkez Osmaniye, Turkey; b Centre for English Language Education, University of Nottingham, Ningbo, 315100, China; c School of Foreign Languages, Soochow University, Suzhou, 215006, China; d School of City Culture and Communication, Suzhou City University, Suzhou, 215104, China

**Keywords:** L2 writing, English modal verbs, L1 influence, Topic effects, Bayesian probabilistic analysis

## Abstract

Modal verbs, with their multifaceted semantic nuances and varied grammatical configurations, present notable challenges for L2 learners and regularly intrigue L2 researchers. This study attempts to investigate and compare how English modal verbs are used by L2 learners from different L1 backgrounds. By exploring the Turkish and Chinese learners’ subcorpora of the International Corpus of Learner English (ICLE), this work scrutizes the overall frequencies of nine core English modal verbs as grouped into three major semantic classes along with the influential lexico-syntatic variables that are semantic classes of collocated verbs, grammatical patterns and subject pronominality. The results of a Bayesian probabilistic analysis show that both the Turkish and Chinese learners primed similar modal verbs and constructional preferences without topic as the normalizing factor. While the broader analysis reveals no statistically significant divergences between these two learner groups in English modal verb preferences, a pronounced contextual influence is evident when the dataset narrows to essays on a unified theme. This nuanced shift underscores the intricate relationship between essay topics and linguistic structures, thus emphasizing the pivotal role of context in modal verb usage.

## 1 Introduction

Modality, an aspect of language that allows speakers to express their attitudes towards the potentiality of a state of affairs [1], plays a crucial role in conveying the degree of truth or reality. The semantic structures of modal verbs are rich and versatile, allowing them to construct genre-specific meanings. For instance, the English modal verb ‘can’ is commonly used to express possibility in technical texts and ability in non-domain specific ones [2]. However, this semantic flexibility, coupled with various possible grammatical constructions, makes English modal verbs challenging for English as a Second Language (L2) or English as a Foreign Language (EFL) learners, particularly those whose first language has a different modal system [[3], [4], [5]]. For instance, considering the modal verb ‘might’, different grammatical constructions highlight its ability to convey various modal nuances. In the ‘Modal + Base Verb’ structure (Modal + Verb_inf_), the phrase ‘might go’ exemplifies how ‘might’ can suggest the potential or possibility of an action within the context of active voice and unmarked aspect. Moving to the ‘Modal + Be + Past Participle Verb’ pattern (Modal + Be + Verb_pp_), the phrase ‘might be seen’ illustrates the passive possibility, where an action or state could be perceived by others, yet the agent remains unspecified. The construction ‘Modal + Have + Past Participle Verb’ (Modal + Have + Verb_pp_), as seen in ‘might have gone’ introduces a speculative reflection on past action, infusing the sentence with a sense of uncertainty about whether the action occurred. In the ‘Modal + Be + Present Participle Verb’ form (Modal + Be + Verb_ing_), exemplified by ‘might be going’, ‘might’ conveys the ongoing yet uncertain nature of an action, blending possibility with the progressive aspect. Finally, the ‘Modal + Have + Been + Past Participle Verb’ structure (Modal + Have + Been + Verb_pp_), demonstrated by ‘might have been done’, employs ‘might’ to speculate about the completion of an action in the past, suggesting that while the action was possible, its actual occurrence remains in doubt. Through these examples, ‘might’ showcases its versatility in expressing possibilities, speculative thoughts, and uncertainties across a range of grammatical contexts, including active and passive voices and perfect and progressive aspects. An understanding and appreciation of these points is clearly important for both students and teachers because, from a communicative language perspective, alongside a test orientated one, misuse or misunderstaidng concerning potential use can have detrimental consequences on personal interactions and achievement.

Previous research has often explored the frequency of modal verbs in L2 writing [[6], [7], [8]]. Although these studies have provided valuable insights into the acquisition of modal verbs by L2 learners, identifying phenomena such as overuse, underuse, and misuse, they often focus primarily on frequency, thereby neglecting the semantic and syntactic functions of these verbs. English modal verbs are context-dependent, with their usage varying according to the genre or topic of the writing prompts. Furthermore, learners' first language (L1) can significantly influence their language use in L2 writing [9]. This study aims to move beyond the mere frequency-based analysis of modal verbs in L2 writing and examine them from both a semantic and syntactic perspective. It also aims to investigate how modal verb usage is affected by writing topics and the learners’ L1. Specifically, this study analyzes English modal verbs in L2 writing by categorizing them into semantic classes and examining their syntactic patterns and subject associations in addition to the semantic classification of lexical verbs used in the pattern. This approach is further enhanced by analyzing the influence of essay topics as contextual determinants, providing a layered understanding of modal verb usage across different semantic purposes, structural contexts, and thematic domains. By integrating thematic considerations, the research offers insights into the dynamic interplay between topic-driven discourse functions and modal verb selection, thereby enriching our understanding of modality in L2 writing through a comprehensive lens encompassing semantic, syntactic, and contextual dimensions by comparing English modal use of learners with two different L1 backgrounds, namely Turkish and Chinese, each language structuralizing modality in idiosyncratic ways. The findings should be of interest to a wide range of readers, ranging from linguists to practitioners, students to course designers.

## 2 Literature review

### 2.1 Theoretical classifications of English modal verbs

The precise semantics and categorization of English modal verbs remain contested. Quirk et al. [10] classify English modal verbs into one of two categories, namely “central and marginal modals” (p. 135) based on modal phrase formation. Central modals (e.g., ‘can’, ‘will’, and ‘must’) precede bare infinitives and serve exclusively finite functions, while marginal modals like ‘dare’, ‘ought’, and ‘need’ necessitate the use of ‘to’ and operate in nonfinite positions. They also introduce intermediary categories like “modal idioms, semi-auxiliaries, and catenatives” (p. 137). Palmer [11] introduces a typological framework for modality, distinguishing between types (“epistemic, deontic, and dynamic”) and degrees (“possibility and necessity”) (p. 36). Epistemic pertains to possibility and prediction, deontic to obligation and permission, and dynamic to ability and volition, including agent intent. Huddleston and Pullum [12] extend Palmer's [11] model by adding a “strength” dimension, spanning from speaker commitment to situation factuality, delineating between necessity (“strong commitment”) and possibility (“weak commitment”) (p. 175). Biber et al. [13] parallel Quirk et al. [10] by segregating English modals into modals and semi-modals. They also demarcate three semantic categories for modals, echoing Palmer's [11] distinctions: the first with verbs like ‘can’, ‘could’, ‘may’, and ‘might’, indicating permission or ability; the second expressing obligation or necessity with verbs like ‘must’ and ‘should’; and the third encompassing volition or prediction with ‘will’, ‘would’, and ‘shall’.

The possible syntactic patterns of English modal verbs are also a topic of heated discussion. Palmer [14] clarifies the syntactic uniqueness of English modal verbs, distinguishing them from lexical verbs through their consistent pre-verbal positioning within clauses, their invariance across different subjects without inflectional modifications [15], and their distinctive subject-auxiliary inversion in interrogative forms [16]. Similarly, Coates [17] observes that these syntactic features serve modals' grammatical functions and play a key role in conveying subtle speaker attitudes, relational dynamics, and pragmatic underpinnings in discourse. The syntactic irregularities of modal verbs, such as their inability to stand alone without a main verb in the bare infinitive form—a phenomenon documented by Huddleston and Pullum [12] and Quirk et al. [10]—are instrumental in their capacity to articulate the specialized modal semantics encompassing possibility, obligation, and necessity, a concept further explored by Palmer [14]. Again, Palmer [14] acknowledges that the hierarchical sequencing observed when multiple modals co-occur in a clause, reflecting a semantic gradation from possibility to necessity, underscores the semantic precision these syntactic constructions afford. Moreover, Biber et al. [13] highlight the syntactic maneuver of inversion in conditional sentences involving modal verbs, a feature that adds to the syntactic repertoire of modal constructions. Depraetere and Reed [15] delve into the complexities of perfect infinitives combined with modals, showcasing how these structures can intricately express nuances of tense and aspect from a syntactic standpoint. Additionally, Huddleston and Pullum [12] note that the syntactic formulation of negation with modal verbs, exemplified in constructions like ‘must not’, presents another layer of syntactic intricacy associated with modals. In synthesizing these perspectives, it becomes evident that the literature extensively documents the multifaceted syntactic frameworks of modal verbs, which are intrinsically tied to their functionality in discourse, their pragmatic utility, and their semantic expressiveness, as supported by seminal works in the field [[16], [17], [18], [19]]. The interplay between the syntactic configurations of modal verbs and their semantic and pragmatic roles illustrates a deep-seated alignment between form, function, and meaning, highlighting the integral role of syntax in the effective deployment of modal verbs within English discourse.

### 2.2 Empirical studies of the use of English modal verbs in L2 writing

An extensive corpus of research delves into the ways learners' first language backgrounds intricately mold their approach to learning and employing modal verbs in L2 English. These studies emphasize the multifarious influence of first language characteristics—spanning syntax, morphology, semantics, pragmatics, and cultural norms—on acquiring and applying modal verbs in an L2 setting [20,21]. For instance, investigations have revealed that learners from a Chinese linguistic background tend to disproportionately favor high-obligation modals such as ‘must’, ‘should’, and ‘have to’, uncovering a pragmatic disconnect in their modal verb deployment, which might stem from cross-linguistic semantic discrepancies or cultural divergences in expressing necessity or advice [22]. Similarly, studies focusing on Arabic-speaking L2 learners have cataloged recurrent challenges and inaccuracies in modal verb selection and usage, revealing a potential misalignment with the semantics and pragmatic uses of modals in English, possibly attributable to the absence of direct modal equivalents in Arabic. Research involving Malaysian learners who navigate a linguistic landscape devoid of direct modal counterparts highlights the formidable hurdles they face in recognizing, let alone accurately employing, English modal verbs, pointing to significant cross-linguistic conceptual gaps [23]. Further research explored how L1 influences cognitive processing and attunement to verbal nuances, affecting learners' ability to discern and interpret modal verbs in English [24]. The scope and depth of modal meanings that learners internalize are also shaped by L1 transfer effects, which can either facilitate or hinder the mapping of modal concepts between L1 and English [25]. Some studies have observed that learners may strategically leverage their L1 knowledge to navigate or comprehend modal meanings in L2, although this reliance varies depending on contextual factors and individual learning trajectories [26]. Collectively, these investigations paint a comprehensive picture of the profound and multifaceted impact of L1 on all facets of L2 modal verb mastery, encompassing understanding, usage, precision, fluency, and metalinguistic insight. This widespread influence stresses the imperative for educators to devise pedagogical strategies that are sensitively attuned to the specific challenges and leverage points presented by learners' L1 backgrounds, thereby fostering more effective and nuanced modal verb competence in L2 English [20,21,[23], [24], [25], [26], [27], [28], [29]].

The complexities of English modals, influenced by socio-cultural constructs in learners' L1 contexts, contribute to pedagogical challenges and learners' restricted modal usage in diverse contexts. Hinkel [3] explicitly illustrates the significant influence of essay topics as a crucial component of context, affecting modal verb usage in academic writing. Topics provide a semantic ground that guides students' choices of modal verbs, aligning with content-specific conventions and engaging with the pragmatic context, requiring students to align their modal verb use with rhetorical norms pertinent to the subject matter. For instance, essays on scientific subjects tend to elicit a higher frequency of logically oriented modals such as ‘must’, reflecting the need for definitive assertions, whereas literary discussions might favor more tentative modals like ‘may’, allowing for ambiguity and interpretation. Moreover, Hinkel's [3] research sheds light on how the nature of the essay—whether argumentative or descriptive—can influence both the frequency and diversity of modal verb use. This variation underscores the interplay between topic and pragmatic considerations, with argumentative texts prompting more frequent yet constrained modal usage compared to the broader modal range employed in descriptive or literary essays. Hinkel [30] further extends the concept of context to include the profound impact of cultural values on modal verb usage. In modal verb acquisition, the blend of social relationships, norms, values, and conventions manifests in the learners' preferences for levels of directness or indirectness and the observance of social hierarchies, which influence modalities of politeness and formality. That is, the divergent modal usage patterns between the learner groups relate to culture-specific influences on academic style, epistemic stance, and linguistic politeness.

Turkish learners' use of English modal verbs and that of Chinese learners pose an intriguing field of inquiry in that their first languages have sharply different modal verb features. The Turkish language marks modality through bound morphemes like ‘-malı’ (‘should’) and ‘abil-’ (‘can’) affixed to verbs and has subject-verb agreement rules like English [31]. In contrast, modality in the mandarin Chinese is expressed through lexical modal verbs like ‘yinggai’ (‘should’) and ‘keyi’ (‘may’) without inflections [22,32]. As noted by Zhao [33], Chinese lacks an explicit grammatical marking of modality, which may lead learners to neglect formal features of English modals. Additionally, improper modal use by Chinese learners may stem from negative L1 transfer, given divergent meaning potentials [34]. Therefore, Turkish learners may benefit from L1 transfer of agreement and bound morphemes, resulting in a positive transfer [31,35,36]. For intstance, Civelek and Karatepe [37] find that Turkish learners use the same modals irrespective of context, suggesting a different understanding of modals, whereas Yang [21] reveal that Chinese learners tend to overuse certain modal verbs like ‘can’, ‘will’, and ‘would’ while underusing ‘may’, thus indicating distinct usage patterns influenced by their respective L1.

Cultural nuances also influence Turkish and Chinese learners' use of English modals in grammatical constructions, modal preferences, and pragmatic interpretations. For example, Turkish learners prefer empirical modality like ‘can’ and ‘would’ which map onto evidentiality marking in Turkish for deduction and hearsay [38]. Contrastively, Chinese learners tend to overuse subject-verb inversion with modals in academic writing, reflecting Chinese conventions for explicit sentence focus [39]. In modal preferences, Chinese learners favor epistemic modality like ‘will’ and ‘must’ to express certainty and authority, aligned with Chinese academic writing norms [30]. Regarding pragmatics, Turkish learners' modal politeness strategies reflect Turkish sociocultural values of social harmony and hospitality [40], whereas Chinese learners interpret modals more literally rather than for mitigating face-threats, stemming from Chinese cultural norms of directness [41].

Despite the myriad of approaches in previous studies, a more granular analysis of English modals in learner data remains crucial. This study, building on the aforementioned factors, zeroes in on the modal usage of Turkish and Chinese learners, examining the probabilistic grammar, constructional predictability, pronominality of subjects, semantic classifications, and collocated verbs' semantic classes. Methodologically, it is based on Biber et al. [13] which, distinguished by its data-driven approach, offers an in-depth examination of English modal verbs employed by native speakers in a broad spectrum of registers to capture the distinctive usage patterns that emerge across diverse communicative contexts. While the analysis does not explicitly address the L2 use of modals, their detailed profiling of modal verb usage is a reference point for the current investigation. Following Biber et al. [13], this study's classification of modal verbs—coupled with the semantic categorization of the lexical verbs they collocate with—forms the groundwork for understanding the modal use in English. This includes insights into the probabilistic grammar underlying modal usage, which refers to the likelihood and predictability of certain modal constructions occurring in specific contexts, as well as the constructional predictability that expresses the regularity and patterned nature of modal verb combinations. Furthermore, the concept of pronominality of subjects, which examines the relationship between the subjects of sentences and the selection and usage of modal verbs, is crucial for understanding how different subject forms—whether pronouns or nouns—affect modal verb choice and application. The semantic classifications provided by Biber et al. [13] note the various meanings and functions that modal verbs and their collocated verbs can convey, ranging from necessity and possibility to permission and ability. In addition, this study also follows Hinkel's [3] approach to probe the potential influence of writing topics on modal constructions, especially in light of L1 differences.

## 3 Methodology

### 3.1 Research questions

This study intends to answer two research questions.

RQ 1 How are core English modal verbs distributed in the argumentative writings of Turkish and Chinese learners respectively in terms of semantic classification, semantic classes of collocated verbs, pattern types and pronominality of the subject?

RQ 2 Do L2 learners' L1 and the topic of choice affect the distribution of English modal verbs and influential variables primed with these modals?

### 3.2 Learner corpora

The study scrutinizes the International Corpus of Learner English (ICLE) Version 3 [42], a collection of English argumentative essays written by L2 learners of different L1s. In particular, this study is focused on the Turkish and Chinese subcorpora of ICLE Version 3, the details of which are presented in Table 1.

**Table 1:** Breakdown of the corpora.

Corpora	Number of essays	Number of words	Average length	Minimum length	Maximum length	Q1	Median	Q3
ICLEv3	9529	5,766,522	605.16	69	4239	488	550	675
Turkish	280	200,601	716.43	500	1423	580.75	716	826
Chinese	982	493,080	502.12	138	1151	436	502	553.75

Q1 and Q3 are interquartile range. The lower quartile or the first quartile (Q1) is the value under which 25.00% of data points are found when arranged in increasing order. The upper or third quartile (Q3) is the value under which 75.00% of data points are found when arranged in increasing order.

Since this study also focuses on the effect of essay topics, both subcorpora are subset to extract essays whose title includes the keyword ‘abortion’, which is the only shared title. It is necessary to point out that the title is the prompt provided to students as the essay topic. Details of the two subsets are presented in Table 2.

**Table 2:** Breakdown of the two subset data.

Sub corpora	Number of essays	Number of words	Average length	Minimum length	Maximum length	Q1	Median	Q3
Turkish	15	11,820	788	500	1009	721.50	716	826
Chinese	71	39,241	552.69	319	933	499.50	548	596.50
Total	86	51,061	605.16	547	4239	488	804	872.00

Q1 and Q3 are interquartile range. The lower quartile or the first quartile (Q1) is the value under which 25.00% of data points are found when arranged in increasing order. The upper or third quartile (Q3) is the value under which 75.00% of data points are found when arranged in increasing order.

### 3.3 Data extraction, classification and analysis

For the data extraction, the study utilized statistical dependency parsing via the Python programming language with the Spacy package [43]. Different from conventional Part-of-Speech (POS) tagging methods, dependency parsing is a head-bound process that relies on dependencies among constituents and relationships between phrases and sentences to define grammatical structures. Taking the finite verb into the center, each lexical unit was defined as a node, and the direct relationship between the head node and child nodes is revealed in a network of hyperlinks. Dependency parsing provides superior results without having to define an n-gram range for each item. In other terms, pattern analysis based solely on POS tagging is vulnerable to failures in observing patterns where root-component dependencies are disrupted by additional lexical items. In natural languages, a given pattern does not have to obey a defined order of query, as a simple pattern such as ‘modal-followed-by-verb’ can also consist of ‘modal-followed-by-adverb-followed-by-verb’. In such cases, POS-based pattern analysis simply disregards a second usage, providing inaccurate observations if all possible constructions are not defined in the query. Therefore, five major patterns suggested by Mindt [44] were defined with dependency rules for the extraction of modal sentences from the corpus.

➢ **Pattern 1 (Modal + Verb**_**inf**_**):** Subject + Modal (present and past forms) + Verb (bare infinitive) – Active Voice, Unmarked Aspect
➢ **Pattern 2 (Modal + Be + Verb**_**pp**_**):** Subject + Modal (present and past forms) + Be + Verb (past participle) – Passive Voice, Unmarked Aspect
➢ **Pattern 3 (Modal + Have + Verb**_**pp**_**):** Subject + Modal (present and past forms) + Have + Verb (past participle) – Active Voice-Perfect Aspect
➢ **Pattern 4 (Modal + Be + Verb**_**ing**_**):** Subject + Modal (present and past forms) + Be + Verb (progressive) – Active Voice, Progressive Aspect
➢ **Pattern 5 (Modal + Have + Been + Verb**_**pp**_**):** Subject + Modal (present and past forms) + Have + Been + Verb (past participle) – Passive Voice, Perfect Aspect

Since this study was focused on the modal structures only, *if* clauses were excluded from the results. Following pattern extraction, subjects were grouped into two as nouns including proper and common nouns and pronouns. Next, verbs and modals were placed into semantic classes suggested by Biber et al. [13]. The semantic classification of verbs consists of seven different groups which are “activity verbs, existence or relationship verbs, mental verbs, communication verbs, facilitation or causation verbs, occurrence verbs, and aspectual verbs” [13, p. 361]. Similarly, modals were grouped into three major semantic classes with the first group “permission/possibility/ability” consisting of ‘can’, ‘could’, ‘may’, and ‘might’, the second group “obligation/necessity” including ‘must’ and ‘should’, and the final group “volition/prediction” consisting of ‘will’, ‘would’, and ‘shall’ [13, p. 485].

Through analysis of modal patterns in the learner data, the study investigated the frequencies of nine core modals, their patterns, voices and aspectual preferences, the status of subject pronominality, and the semantic classes of verbs collocated with these modals. Furthermore, modals were classified into three semantic groups as suggested by Biber et al. [13]. Finally, it scrutinized whether the essay topic influenced the priming of certain English modals and modal constructions. Therefore, two datasets were prepared for the study. The first one included all the modal sentences from both subcorpora for exploratory data analysis. For the second dataset, however, with a focus on contextual factors, modal sentences from essays with a common topic in both subcorpora were subset and applied to a second round of regression analysis.

**Fig. 1:**
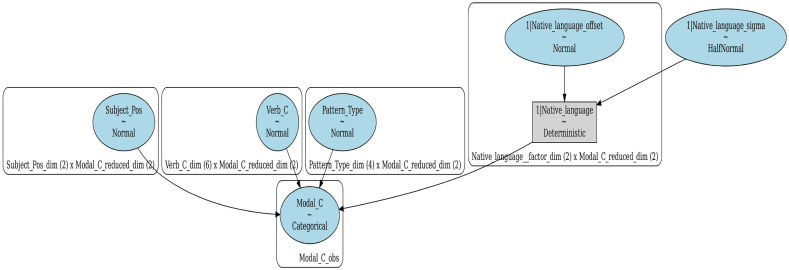
Visualization of the regression model.

**Fig. 2:**
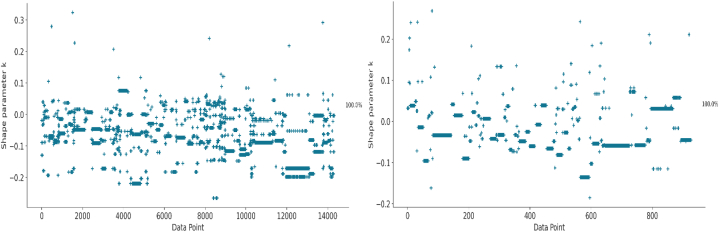
Estimated paretoshape parameters for diagnosing convergence.

In the actual operation, this study employed a Bayesian categorical (multinomial) regression model to examine the relationship between lexical, grammatical, and contextual factors with the odds of observing modals grouped into three semantic classes. The model was fit using the PyMC4 [45] and Bambi [46] packages in Python with weakly informative priors (population mean ‘mu’ = 0, sigma = 1). The model included semantic classes of modals as the response variable and semantic classes of verbs, subjects' POS tags, and pattern types as predictor variables, in addition to the learners' first language as a random effect variable (see Fig. 1). The same model was applied a second time on the subset data consisting of only texts whose titles include the keyword ‘abortion’, a topic shared in both datasets. As for the model validation and performance evaluation, Pareto-smoothed importance sampling leave-one-out cross-validation (PSIS-LOO-CV) was computed [47] (see Fig. 2).

➢ **Native language (a factor with two levels):** Turkish and Chinese
➢ **Subject POS tags (a factor with two levels):** Noun and pronoun
➢ **Semantic classes of modals (a factor with three levels):** Obligation/necessity (Oblig_Necess), permission/possibility/ability (Perm_Poss_Abil), volition/prediction (Volit_Predict)
➢ **Semantic classes of verbs (a factor with seven levels):** Activity verbs, mental verbs, existence or relationship verbs, facilitation or causation verbs, occurrence verbs, communication verbs, and aspectual verbs
➢ **Pattern types (a factor with five levels):** Modal + Verb_inf_, Modal + Be + Verb_pp_, Modal + Have + Verb_pp_, Modal + Be + Verb_ing_, Modal + Have + Been + Verb_pp_

## 4 Results and discussion

### 4.1 Frequencies, semantic classes and syntactic patterns of English modal verbs in L2 writing

Table 3 presents the frequency distribution of modal verbs for both the comprehensive dataset and the specific ‘abortion’ dataset, juxtaposing the semantic class ratios of Turkish and Chinese learners. The initial set of modal verbs—comprising ‘can’, ‘could’, ‘may’, and ‘might’—articulates notions of permission, possibility, or ability. The subsequent set, encompassing ‘must’ and ‘should’, conveys a sentiment of obligation or necessity. The final cohort, which incorporates ‘will’, ‘would’, and ‘shall’, delineates expressions of volition or prediction.

**Table 3:** Overall frequencies of selected modal verbs in two datasets across subcorpora.

Datasets	Modal verbs	L1s					
		Turkish			Chinese		
		Frequency	Percent (%)	Within class percent (%)	Frequency	Percent (%)	Within class percent (%)
Total dataset	Can	1435	40.70	79.98	3940	36.50	65.49
	Could	92	2.61	5.12	436	4.04	7.24
	May	221	6.27	12.31	1542	14.30	25.63
	Might	46	1.30	2.56	98	0.90	1.62
	Must	212	6.01	22.22	281	2.60	19.37
	Shall	3	0.08	0.38	14	0.13	0.41
	Should	742	21.00	77.77	1169	10.80	80.60
	Will	650	18.40	83.65	2191	20.30	65.61
	Would	124	3.52	15.95	1132	10.50	33.96
	**Total**	**3525**	**100**		**10805**	**100**	
‘Abortion’ dataset	Can	84	48.27	77.77	257	34.08	66.23
	Could	15	8.62	13.88	41	5.43	10.56
	May	8	4.59	7.40	85	11.27	21.90
	Might	1	0.57	0.92	5	0.66	1.20
	Must	3	1.72	10.71	19	2.51	15.44
	Shall	1	0.57	2.63	2	0.20	62.13
	Should	25	14.36	89.28	104	13.79	84.55
	Will	28	16.09	73.68	151	20.02	62.13
	Would	9	5.17	23.68	90	11.93	37.03
	**Total**	**174**	**100**		**754**	**100**	

Table 3 delineates the modal verb frequencies for the Turkish and Chinese subcorpora. The Turkish dataset encompasses 3,525 modal occurrences, constituting approximately 1.75% of its total lexemes. Conversely, the Chinese dataset documents 10,805 modal verb instances, representing 2.19% of its comprehensive word count. This data underscores a marginally augmented presence of English modal verbs within the Chinese learners’ texts.

The modal class labeled as ‘Perm_Poss_Abil’, encompassing ‘can’, ‘may’, ‘could’, and ‘might’, is prominently represented in both datasets, accounting for a majority of all modal usages. However, divergent patterns emerge for the subsequent classes. Specifically, the Chinese data allocates roughly 31.00% of its modals to the ‘Volit_Predict’ category, which integrates ‘will’, ‘would’, and ‘shall’. In comparison, the Turkish data predominantly leans towards the ‘Oblig_Necess’ category, featuring ‘should’ and ‘must’, representing approximately 27.00% of its modal composition. The latter category also resonates in the Chinese dataset but at a reduced frequency of 13.90%.

A detailed analysis of individual modal verbs underscores ‘can’ as the paramount modal in both datasets, with ‘shall’ registering minimal occurrences. Within the Turkish learner dataset, ‘can’ dominates with 1,435 instances (or 40.70%), followed by ‘should’ with 742 occurrences (or 21.00%). For the Chinese learners, ‘can’ features most prominently with 3,940 instances (or 36.50%), followed by ‘will’ with 2,191 occurrences (or 20.30%). Notably, ‘might’ is the least represented modal for both groups, registering 46 instances (or 1.30%) for the Turkish learners and 98 instances (or 0.90%) for the Chinese learners. Other disparities include the elevated prominence of ‘should’ in the Turkish dataset, ranking second, while it assumes a fourth placed prominence ranking in the Chinese data. Additionally, ‘must’ is 6.00% more frequent in the Turkish dataset compared to a mere 2.00% in the Chinese one.

For the ‘Perm_Poss_Abil’ category, ‘can’ remains predominant for both linguistic groups. Within the ‘Oblig_Necess’ category, ‘should’ surpasses ‘must’ in frequency. However, a stark contrast emerges: in the Chinese dataset, ‘should’ is nearly eight times as frequent as ‘must’, whereas in the Turkish dataset, it is roughly four times as prevalent. Within the ‘Volit_Predict’ domain, ‘will’ is the foremost modal, succeeded by ‘would’ and ‘shall’. In the Chinese subset, 66.00% of structures containing a modal from this category feature ‘will’, while this proportion ascends to 84.00% in the Turkish subset.

Further examination of a focused dataset on ‘abortion’ reveals congruent modal frequency patterns in both linguistic datasets. The Turkish subset on ‘abortion’ consists of 174 modal instances, while the Chinese counterpart documents 754 instances. Intriguingly, ‘can’ persists as the most recurrent modal verb for both datasets, with 84 occurrences (or 48.27%) in the Turkish subset and 257 occurrences (or 34.08%) in the Chinese subset. While both linguistic groups exhibit overlapping modal verb preferences, distinct nuances in their frequency distributions and modal hierarchies are discernible. This systematic and highly detailed analysis offers an insightful comparative lens into the modal verb dynamics among the Turkish and Chinese learners.

Table 4 underscores that the pattern ‘Modal + Verb_inf_’, indicative of the simple aspect, is the predominant structure affiliated with core English modal verbs. Remarkably, this pattern constitutes 90.00% of all sentences featuring a modal verb in both datasets. The subsequent prevalent structure is ‘Modal + Be + Verb_pp_’. Among the patterns, the modal with the perfect aspect in passive voice emerges as the least favored by both learner groups. Notably, the syntactic patterns exhibit a pronounced similarity between the two groups. This alignment extends to the subset data, where comparable patterns consistently feature.

**Table 4:** Overall frequencies of modal patterns in two datasets across subcorpora.

Datasets	Patterns	L1s			
		Turkish		Chinese	
		Frequency	Percent (%)	Frequency	Percent (%)
Total dataset	Modal + Verb_inf_	3173	90.00	9753	90.30
	Modal + Be + Verb_pp_	311	8.82	792	7.33
	Modal + Have + Verb_pp_	28	0.79	156	1.44
	Modal + Be + Verb_ing_	11	0.31	96	0.88
	Modal + Have + Be + Verb_pp_	2	0.05	8	0.07
	**Total**	**3525**	**100**	**10805**	**100**
‘Abortion’ dataset	Modal + Verb_inf_	153	87.93	684	90.71
	Modal + Be + Verb_pp_	16	9.19	56	7.42
	Modal + Have + Verb_pp_	2	1.14	10	1.32
	Modal + Be + Verb_ing_	2	1.14	4	0.53
	Modal + Have + Be + Verb_pp_	1	0.50	–	–
	**Total**	**174**	**100**	**754**	**100**

Table 5 illustrates a divergent preference between noun and pronoun subjects in modal sentences across the two learner groups, in contrast to the marked similarities in their grammatical patterns. Speficially, the Turkish subcorpus exhibits a pronounced preference for pronouns, accounting for 59.30%, with nouns trailing at 40.70%. In contrast, the Chinese learners’ subcorpus displays a slight inclination towards nouns at 52.60%, compared to pronouns at 47.40%. Interestingly, however, the subset data presents a shift: nouns are more frequently used by the Turkish learners, while the Chinese learners favor pronouns, deviating from the trends observed in the comprehensive dataset. When compared across both learner groups, this divergence may offer preliminary evidence of contextual influences on modal constructions (see Table 6).

**Table 5:** Pronominality of subjects in modal constructions in two datasets across subcorpora.

Datasets	Subject POS-Tag	L1s			
		Turkish		Chinese	
		Frequency	Percent (%)	Frequency	Percent (%)
Total dataset	Noun	1433	40.70	5679	52.60
	Pronoun	2092	59.30	5126	47.40
	**Total**	**3525**	**100**	**10805**	**100**
‘Abortion’ dataset	Noun	114	65.51	346	45.88
	Pronoun	60	34.48	408	54.11
	**Total**	**174**	**100**	**754**	**100**

**Table 6:** Semantic classes of verbs collocated with modals in two datasets across subcorpora.

Datasets	Semantic classes of verbs	L1s			
		Turkish		Chinese	
		Frequency	Percent (%)	Frequency	Percent (%)
Total dataset	Activity verbs	1252	35.00	4110	38.00
	Mental verbs	712	20.20	2016	18.70
	Existence or relationship verbs	825	23.40	1750	16.20
	Facilitation or causation verbs	212	6.00	1420	13.10
	Occurrence verbs	136	3.86	702	6.50
	Communication verbs	307	8.71	638	5.90
	Aspectual verbs	81	2.30	169	1.56
	**Total**	**3525**	**100**	**10805**	**100**
‘Abortion’ dataset	Activity verbs	72	41.37	293	38.85
	Mental verbs	29	16.66	141	18.70
	Existence or relationship verbs	30	17.24	113	14.98
	Facilitation or causation verbs	15	8.62	95	12.59
	Occurrence verbs	9	5.17	44	5.83
	Communication verbs	11	6.32	52	6.89
	Aspectual verbs	8	4.59	16	2.12
	**Total**	**174**	**100**	**754**	**100**

In examining semantic verb classes collocated with core English modals, activity verbs consistently emerge as the most prevalent, while aspectual verbs rank lowest in both datasets. Nevertheless, distinctions become apparent in the other categories. The Turkish subcorpus features 23.40% of structures with existence or relationship verbs, compared to approximately 16.00% in the Chinese subcorpus. The Chinese dataset demonstrates a heightened preference for facilitation or causation verbs and occurrence verbs. Conversely, the Turkish learners exhibit a marked inclination towards mental verbs and communication verbs. Analyzing the subset data reveals nuanced shifts: the Turkish dataset records an increase in the proportions of activity verbs and aspectual verbs, contrasted against a decline in mental verbs and communication verbs. In parallel, the Chinese subset data manifests a heightened prevalence of aspectual verbs and communication verbs.

### 4.2 L1 influence and topic effects on the use of English modal verbs in L2 writing

This section presents the regression analysis outcomes, which have been elaborately validated as outlined in the methodology. Posterior estimates are primarily conveyed in terms of median, credible interval, and the highest posterior density interval (HDI at 89.00%), except when specified differently. However, only significant values pertaining to the credible interval are elaborated upon here.

The analysis derived estimates from two distinct models, utilizing 4,000 posterior samples and varying observation counts. The inaugural model was predicated on 14,330 observations, while the subsequent model was based on a 928 observation log-likelihood matrix. The first model's expected log predictive density (elpd_loo), a metric gauging the model's predictive efficacy, stood at −13,761.30. Its standard error (SE) 57.35 denoted the imprecision linked with this elpd_loo computation. Furthermore, the model's complexity was inferred from an effective number of parameters (p_loo) of 21.18.

In contrast, the second model registered an elpd_loo of −900.34, reflecting a differentiated predictive prowess relative to the first. This model's SE was cataloged at 14.84, thus manifesting diminished uncertainty vis-à-vis the elpd_loo estimation compared to its predecessor. A p_loo value of 25.04 intimated its heightened intricacy relative to the first model. Convergence was successfully attained for both models, corroborated by the estimated Pareto distribution parameter, which remained below 0.50. Nevertheless, a contrast in their predictive capacities was discernible. The first model's considerably negative elpd_loo value of −13,761.30 versus the second model's −900.34 suggests superior predictive acumen for the latter.

In Table 7, the regression analysis underscores notable associations between varied verb categories and pattern types concerning the outcome variable, specifically under the conditions of permission, possibility, ability, volition, and prediction. Variables of significance, as delineated by credible intervals (HDI = 0.89), are delineated, employing the modal semantic class of obligation and necessity as a benchmark for response. The analysis evinces discernible variations across different modal semantic classes concerning verb semantic classes and pattern types. In the model without an intercept for the predictors, aspectual verbs in the context of permission, possibility, and ability demonstrate a negative relationship with the outcome (Median = −0.41, MAD = 0.11, ETI = [−0.68, −0.15]). Existence or relationship verbs exhibit negative correlations both in permission, possibility, and ability (Median = −0.61, MAD = 0.04, ETI = [−0.71, −0.51]) as well as in volition and prediction conditions (Median = −0.18, MAD = 0.05, ETI = [−0.30, −0.07]). This suggests that the likelihood of observing these modal verbs associated with specific semantic classes is reduced compared to the modals of obligation and necessity.

**Table 7:** Regression coefficients for variables with significance based on credible interval.

Variables	Median	MAD	ETI_5.5	ETI_94.5
Verb_C [Aspectual, Perm_Poss_Abil]	−0.41	0.11	−0.68	−0.15
Verb_C [Communication, Volit_Predict]	0.33	0.08	0.16	0.50
Verb_C [Existence or relationship, Perm_Poss_Abil]	−0.61	0.04	−0.71	−0.51
Verb_C [Existence or relationship, Volit_Predict]	−0.18	0.05	−0.30	−0.07
Verb_C [Facilitation or causation, Perm_Poss_Abil]	0.32	0.06	0.18	0.46
Verb_C [Facilitation or causation, Volit_Predict]	0.37	0.06	0.22	0.53
Verb_C [Occurrence, Perm_Poss_Abil]	0.97	0.10	0.74	1.21
Verb_C [Occurrence, Volit_Predict]	1.25	0.10	1.01	1.50
Pattern_Type [Modal + Be + Verb_ing_, Perm_Poss_Abil]	−1.36	0.15	−1.73	−0.99
Pattern_Type [Modal + Be + Verb_ing_, Volit_Predict]	−0.84	0.17	−1.22	−0.44
Pattern_Type [Modal + Be + Verb_pp_, Perm_Poss_Abil]	−0.96	0.05	−1.09	−0.84
Pattern_Type [Modal + Be + Verb_pp_, Volit_Predict]	−0.91	0.06	−1.05	−0.76
Pattern_Type [Modal + Have + Verb_pp_, Perm_Poss_Abil]	−0.46	0.13	−0.76	−0.15

Verb_C stands for verb semantic class, while MAD is median absolute deviation and ETI is Equal-Tailed Interval (Highest Density Interval = 0.89).

Conversely, existence or relationship verbs present strong positive associations in both aforementioned conditions. Specifically, verbs in the permission, possibility, and ability context are more likely aligned with modals such as ‘can’, ‘could’, ‘may’, and ‘might’. In contrast, they lean more towards modals like ‘will’, ‘would’, and ‘shall’ in the volition and prediction context. Furthermore, occurrence verbs exhibit pronounced positive correlations in both conditions, thereby implying a heightened likelihood of association with modals when contrasted with the reference of obligation and necessity.

However, modal semantic groups across both conditions demonstrate diminished inclination towards patterns relative to the reference level of obligation and necessity. For example, the estimated coefficient for patterns like ‘Modal + Be + Verb_ing_’, ‘Modal + Be + Verb_pp_’, and ‘Modal + Have + Verb_pp_’ suggests a lower likelihood of association with modals of permission, possibility, and ability in contrast to the reference group. A parallel observation is noted with the patterns ‘Modal + Be + Verb_ing_’ and ‘Modal + Be + Verb_pp_’ with the modals of volition and prediction.

The interplay between diverse semantic verb classes and modal semantic classes therefore underscores the pronounced salience of the obligation and necessity reference level with certain verbs compared to other modal semantic classes. Nevertheless, modals such as ‘can’, ‘could’, ‘may’, ‘might’, ‘will’, ‘would’, and ‘shall’ exhibit a higher propensity to pair with specific verbs across varied semantic categories. Concerning pattern preferences, each pattern manifests a higher likelihood with obligation and necessity modals, indicating their versatility across grammatical constructions.

Table 8 elucidates the regression coefficients concerning learners’ native languages. A prominent association emerges solely between learners possessing Chinese as their primary language and the semantic category of permission, possibility, and ability. The deduced regression coefficient suggests that the log-odds of encountering modals within this particular category augmented by 0.90 for the Chinese learners (Median = 0.90, MAD = 0.36, CI [0.16, 1.97]), in contrast to the semantic paradigm of obligation and necessity. This infers a propensity among the Chinese learners to frequently employ modals linked to permission, possibility, and ability over those tethered to obligation and necessity.

**Table 8:** Regression coefficients for learners’ first languages.

Variables	Median	MAD	ETI_5.5	ETI_94.5
Native_language_offset [Chinese, Perm_Poss_Abil]	0.98	0.39	0.18	1.97
Native_language_offset [Chinese, Volit_Predict]	0.72	0.40	−0.08	1.88
Native_language_offset [Turkish, Perm_Poss_Abil]	0.16	0.35	−0.90	0.97
Native_language_offset [Turkish, Volit_Predict]	−0.41	0.38	−1.54	0.40
Native_language_sigma	0.92	0.32	0.43	2.19
**Native_language [Chinese, Perm_Poss_Abil]**	**0.90**	**0.36**	**0.16**	**1.97**
Native_language [Chinese, Volit_Predict]	0.66	0.31	−0.09	1.50
Native_language [Turkish, Perm_Poss_Abil]	0.15	0.36	−0.59	1.23
Native_language [Turkish, Volit_Predict]	−0.38	0.31	−1.13	0.45

Verb_C stands for Verb Semantic Class, while MAD is median absolute deviation and ETI is Equal-Tailed Interval (HDI = 0.89).

Contrastingly, the data for the Chinese learners does not manifest significant disparities pertaining to the semantic group of volition and prediction. Similarly, discernible variations remain absent among the Turkish learners across all modal classifications, as substantiated by the outcomes in the permission, possibility, and ability segment (Median = 0.15, MAD = 0.36, CI [−0.59, 1.23]) and the volition and prediction segment (Median = −0.38, MAD = 0.31, CI [−1.13, 0.45]). This suggests a consistent modal usage by the Turkish learners across diverse semantic classes, indicating that their linguistic foundation minimally influences their modal selection within these domains.

It can thus be suggested that while the Chinese learners exhibit a discernible inclination towards modals associated with permission, possibility, and ability as opposed to obligation and necessity, the Turkish learners demonstrate equanimity in their modal usage across varied semantic domains.

Table 9 delineates the regression coefficients for pertinent variables within the refined dataset, encompassing essays unified by title. Subsequent to this data refinement, the coefficients undergo marginal modifications. A salient observation indicates that when compared to the modal category of obligation and necessity, the log-odds of subjects manifesting as pronouns escalate by an average of 1.51 (Median = 1.52, MAD = 0.30, CI [0.32, 2.05]) within the modals associated with permission, possibility, and ability. Moreover, in this streamlined dataset, the volume of variables exerting significant effects diminishes. For the quartet of residual variables encapsulated in Table 9, the impacts mirror those discerned in the antecedent model. In particular, the propensity to identify existence or relationship verbs conjoined with modals of permission, possibility, and ability recedes (Median = −1.02, MAD = 0.17, CI [−1.42, −0.63]). In contrast, the likelihood of detecting occurrence verbs increases, becoming approximately 3.8 times (exp 1.33) more prevalent alongside modals of volition and prediction compared to their counterparts in the obligation and necessity category (Median = 1.33, MAD = 0.33, CI [0.61, 2.14]). Conclusively, the odds of discerning the grammatical construct ‘Modal + Be + Verb_pp_’ (passive voice, simple aspect) diminishes threefold for both modal groups relative to those in the obligation and necessity category. This trend is evident both for modals of permission, possibility, and ability (Median = −1.00, MAD = 0.19, CI [−1.45, −0.52]) and those of volition and prediction (Median = −1.08, MAD = 0.22, CI [−1.60, −0.56]).

**Table 9:** Regression coefficients for variables with significant effects on reduced data.

Variables	Median	MAD	ETI_5.5	ETI_94.5
Subject_Pos [PRON, Perm_Poss_Abil]	1.51	0.30	0.32	2.05
Verb_C [Existence or Relationship, Perm_Poss_Abil]	−1.02	0.17	−1.42	−0.63
Verb_C [Occurrence, Volit_Predict]	1.33	0.33	0.61	2.14
Pattern_Type [Modal + Be + V_pp_, Perm_Poss_Abil]	−1.00	0.19	−1.45	−0.52
Pattern_Type [Modal + Be + V_pp_, Volit_Predict]	−1.08	0.22	−1.60	−0.56

Verb_C stands for Verb Semantic Class, while MAD is median absolute deviation and ETI is Equal-Tailed Interval (HDI = 0.89).

**Fig. 3:**
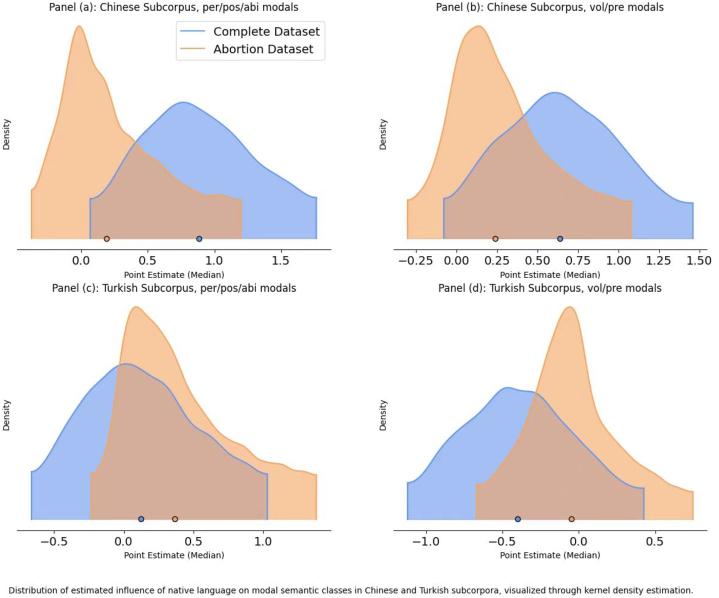
Estimated coefficients for semantic classes of modals per native language across two datasets.

Fig. 3 delineates the coefficients of modal semantic classes stratified by each learner group's native language across dual models. When controlling for other variables, the modal categories of permission, possibility, ability, volition, and prediction manifest diminished probabilities in the ‘abortion’ data for the Chinese dataset compared to the obligation and necessity classes. In contrast, these modal categories exhibit a heightened occurrence in the Turkish dataset's ‘abortion’ data. Notably, these categories also showcase increased probabilities in the Chinese dataset vis-à-vis the Turkish dataset. However, these discrepancies are not statistically significant within the HDI of 0.89.

Initial observations indicate a marginally amplified modal usage in the Chinese subcorpus. The modal ‘can’ dominated both datasets, with ‘should’ and ‘will’ the next two most prominent terms in the Turkish and Chinese subcorpora, respectively. Consequently, the modal class ‘Perm_Poss_Abil’ (encompassing permission, possibility, and ability), which includes ‘can’, ‘may’, ‘could’, and ‘might’, predominated in both datasets—over half the modal utterances employed these verbs. Essentially, the elevated frequency of ‘can’ spurred this trend. Distinct variations emerged in other semantic classes: the ‘Volit_Predict’ class (comprising ‘will’, ‘would’, and ‘shall’) was accentuated in the Chinese dataset, while the Turkish one favored ‘Oblig_Necess’. Analyzing within-class modal ratios, ‘should’ reigned supreme in the ‘Oblig_Necess’ category for both datasets, overshadowing ‘must’ by factors of eight and four in the Chinese and Turkish subcorpora, respectively. Within the ‘Volit_Predict’ class, ‘will’ was paramount.

Secondly, grammatical pattern preferences remained largely consistent. The pattern ‘Modal + Verb_inf_’ representing a present tense and an active voice was prevalent in both datasets, while ‘Modal + Have + Be + Verb_pp_’ representing a perfect aspect passive voice was least frequent. Regarding subject pronominality, the Chinese dataset exhibited a noun preference, while the Turkish dataset leaned towards pronouns, albeit with marginal differences within each dataset. Analysis spotlighted activity verbs as the predominant semantic class collocated with the core modals. Nevertheless, nuances surfaced: existence or relationship verbs favored the Turkish dataset, whereas the Chinese dataset displayed a disposition towards facilitation or causation verbs and occurrence verbs, and the Turkish dataset towards mental verbs and communication verbs. These observations primarily held ground in the ‘abortion’-centric subset data, maintaining consistency with modal prevalence and distribution.

However, subtle discrepancies emerged between the complete and subset datasets, primarily regarding subject pronominality and verb semantic class frequencies. Contrary to the overarching dataset, the subset displayed a Turkish preference for nouns and a Chinese proclivity for pronouns. This shift might hint at contextual and L1-influenced priming disparities in grammatical inclinations. Furthermore, regarding verb choices collocated with the core modals, the Turkish subset displayed an affinity for activity verbs and aspectual verbs. In contrast, the Chinese subset gravitated towards aspectual verbs and communication verbs. These findings suggest that context shifts, potentially modulated by native language influences, might drive variances in verb usage trends.

### 4.3 Discussion

#### 4.3.1 Adopting a cautious view of the L1 influence on L2 learners’ use of English modal verbs

The current study challenges the prevailing notion that L1 significantly influences the Turkish and Chinese learners' utilization of English modal verbs. Contrary to established perspectives positing a robust L1 influence on L2 modal verb preferences, our findings suggest otherwise. Although there were minor numerical discrepancies, the Turkish and Chinese learners predominantly employed the same modal verbs, notably ‘can’, ‘should’, and ‘will’. However, distinctions arose, such as the heightened predilection for ‘must’ in the Turkish subcorpus and a greater inclination towards ‘may’ in the Chinese subcorpus. The regression analysis, exemplified in Fig. 3, corroborates this, revealing negligible differences when considering L1 as a variable.

Nonetheless, when compared with other factors, L1's nuanced influence becomes discernible, albeit marginally. As delineated in Table 8, the Chinese learners demonstrate a 2.45-fold increased likelihood to employ modals from the categories of permission, possibility, and ability compared to other modal categories. However, this trend fails to establish a statistically significant divergence between the two learner cohorts. This hints at the potential influence of L1, albeit predominantly on the lexico-grammatical structures rather than the mere selection of modals.

Further insights emerge from the subset data analysis. Here the modal-pronominal preferences of both groups deviate from both intra-group and inter-group patterns observed in the comprehensive dataset. Table 5 elucidates that, in the overarching dataset, the Turkish learners predominantly utilize pronouns over nouns in modal constructs, a trend absent in their Chinese counterparts. Conversely, the subset data reveals an inversion of this trend: the Turkish learners predominantly employ nouns, while the Chinese learners exhibit a pronoun preference. This nuanced shift underscores the intricate interplay between L1 backgrounds and the conceptual framing of topics, manifesting in the grammatical choices of diverse learner groups. The findings also raise interesting questions about whether changes have occurred in linguistic usage over time in both contexts. In addition, the data also generates thought provoking considerations such as whether teaching practice has evolved in the two national contexts influencing developments, alongside the extent to which access to similar online English information sources may or may not have done so.

#### 4.3.2 Verifying the effects of contextual factors on the L2 use of English modal verbs

This investigation underscores the profound impact of contextual determinants on modal constructions. Both frequency and regression analyses affirm the salient influence of selected variables in these constructions, with the semantic classes of collocated verbs consistently emerging as pivotal, irrespective of the context. Notably, even when the data was narrowed down to a specific topic, certain semantic classes exhibited distinct associations, suggesting context-specific influences. Additionally, context shifts resulted in altered relationships between grammatical patterns and modals classified semantically. A case in point is the pronounced preference for passive voice constructions with modals of obligation and necessity.

Arguably the most pronounced observation pertained to the pronominality of subjects in different contexts. Clear tendencies emerged in noun versus pronoun preferences among both learner groups, revealing both context-specific and L1-influenced variations. For instance, the Turkish learners displayed a propensity for nouns within the ‘abortion’-centric dataset, whereas their Chinese counterparts favored pronouns—a stark contrast to trends observed in the broader dataset. Furthermore, modals related to permission, possibility, and ability in this specific context exhibited a stronger association with pronouns. Thus, constructs such as ‘Pronoun + can/could/may/might’ become more probable when the structure diverges from the ‘Modal + Be + Verb_pp_’ pattern and when the associated verb does not belong to the existence or relationship category. This contextual modulation underlines the imperative for L2 learners to grasp the cultural nuances and their interplay with grammatical constructions, modal preferences, and pragmatic interpretations, enriching their mastery over English modals.

This study has also raised additional awareness of the pronounced effect of essay topics on modal constructs, highlighting robust evidence for the influence of contextual factors on L2 learners' use of English modal verbs. Rather than solely influencing modal verb choices, the topics prominently swayed the lexical verbs, subjects collocated with modals, and the overarching grammatical structures. In the subset data focused on ‘abortion’, an inverse trend was observed: the Turkish learners favored nouns, while the Chinese learners leaned towards pronouns—a divergence from the comprehensive dataset's patterns. The regression analysis further cemented the preeminence of verb choices in shaping modal preferences, revealing certain semantic verb classes as particularly influential in diverse contexts. These observations resonate with Hinkel's [3] assertion that cultural and topic-related factors can profoundly influence modal verb utilization. This intertwining of learners' L1 backgrounds, cultural contexts, and personal experiences suggests a multifaceted interplay, shaping their lexical choices and modal constructs. The implications from these findings are also pertinent. Readers from a similar background will appreciate the writer's intended meaning, but those from different cultural backgrounds may read unintended nuances into the word choices which the writer may not knowingly be applying. This can lead to broader cultural misinterpretations as third country and/or culture readers will assume differences between wirters from the two cultures based on strength of opinion when in fact this may not be the case. In many ways this is similar to rater differences in assigning scores, and the way in which scale interpretation impacts upon assigned mark allocations [48].

Both the frequency and regression analyses align with past research in demonstrating the complex and nuanced role of L1 transfer in L2 acquisition, that semantic domain and collocate verb type have a substantial effect on modal construction, often outweighing the role of L1 background [[49], [50], [51]]. As Ellis [49] notes, L1 transfer should be considered just one factor interacting dynamically with other variables like proficiency level, task type, semantic domain, so on and so forth. The regression analysis provides further evidence of this, with L1 background having a negligible effect on modal choice compared to other predictors like verb type. The usage-based perspectives provide a useful framework for interpreting these findings, emphasizing the role of usage events and conceptual patterns rather than L1 transfer alone in shaping L2 development [49,52]. The study's regression analysis supports this approach, with semantic domain and verb type emerging as more salient predictors of modal choice than L1 background. In particular, the findings on specific semantic classes mirror Tyler's [53] work, revealing the interaction between semantic and grammatical factors in which she observed constructions expressing possibility were primed with dynamic rather than stative verbs. The effects on dynamic versus stative verb collocates also have parallels in Gilquin's [50] corpus analysis revealing probability modals occur more frequently with event verbs. She suggests that this coupling allows speakers to construe events as uncertain or potential rather than actualized. Similarly, subset analysis in the current study showed an increased salience of possibility modals like ‘can’, ‘could’, and ‘might’ when paired with dynamic verbs outside of the existence/relationship domain.

However, the subset analysis of essays on ‘abortion’ revealed intriguing shifts in pronominal preferences and grammatical patterning between learner groups, indicating the interaction between semantic, pragmatic, and grammatical factors in L2 acquisition [53]. The shifting pronominal preferences dependent on the essay topic also relate to research on conceptual priming in L2 production. Studies have shown that priming a particular conceptual domain can trigger learners to reuse associated linguistic structures [54]. The ‘abortion’ essays may have conceptually primed learners to favor either nouns or pronouns based on L1-specific construals. Furthermore, the contextual influence on passive voice constructions with obligation/necessity modals aligns with corpus studies revealing topic and genre effects on this grammatical pattern [13]. Passives are often more frequent in academic proses and topics requiring a detached, impersonal style. For example, the subset analysis revealed shifts in pronominal preferences based on essay topic and L1 background. This reflects findings from learner corpora showing variability in argument structure and information flow patterns according to prompt type and L1 [55]. The ‘abortion’ essays may have conceptually and culturally, and unknowingly, led learners to construe participants as either pronouns or nouns. Overall, the multiple context-sensitive effects empirically confirm the close ties between modal semantics and conceptual structure posited by cognitive linguistic frameworks [52].

## 5 Conclusion

This research offers a meticulous examination of the deployment of nine fundamental modal verbs within the Turkish and Chinese subcorpora of the ICLE. Drawing on three salient semantic classes, pronominality of subjects, verb semantic classes, and five distinct tense-aspect grammatical patterns, the findings underscore comparable modal verb usage patterns and constructional propensities among both the Turkish and Chinese learners. This congruence intimates a minimal role of native language in shaping English modal verb usage. Nevertheless, the investigation also highlights the pronounced influence of writing topics on modal verb deployment and structuring. This denotes that linguistic expressions of modality transcend mere lexical selections and are deeply rooted in discourse context or topic.

However, the study is not without limitations. The dataset's essay count was circumscribed, and the inquiry's purview was narrowly focused on the Turkish and Chinese learners, dictated by spatial considerations. Enriched future studies might encompass essays penned by learners from diverse linguistic backgrounds. Additionally, exploring modality from a broader vantage point, beyond mere modal verb usage, to incorporate other modal expression avenues would be enlightening. Pursuits integrating more variables promise richer, more universally applicable insights, advancing a holistic comprehension of learner language nuances.

## Funding statement

This research was funded by the Humanities and Social Sciences Interdisciplinary Research Team of 10.13039/501100007824Soochow University (Grant No: 5033720623).

## Data availability statement

The data of this research is available at https://github.com/fatihbozdag/Grammar-Research-with-Spacy/blob/main/modal_patterns.

## CRediT authorship contribution statement

**Fatih Ünal Bozdağ:** Writing – original draft, Methodology, Data curation, Conceptualization. **Gareth Morris:** Writing – review & editing. **Junhua Mo:** Writing – review & editing, Writing – original draft, Methodology, Funding acquisition, Data curation.

## Declaration of competing interest

The authors declare the following financial interests/personal relationships which may be considered as potential competing interests: Junhua Mo reports article publishing charges was provided by Suzhou City University. Junhua Mo reports financial support was provided by 10.13039/501100007824Soochow University. If there are other authors, they declare that they have no known competing financial interests or personal relationships that could have appeared to influence the work reported in this paper.
